# Short-Term and Long-Term Effects of Riding for Children with Cerebral Palsy Gross Motor Functions

**DOI:** 10.1155/2018/4190249

**Published:** 2018-07-08

**Authors:** L. Žalienė, D. Mockevičienė, B. Kreivinienė, A. Razbadauskas, Ž. Kleiva, A. Kirkutis

**Affiliations:** ^1^Klaipėda University's Faculty of Health Sciences, Klaipėda LT-92294, Lithuania; ^2^Lithuanian Sea Museum, Klaipėda LT-93100, Lithuania

## Abstract

*Aim*. To evaluate the effects of riding for beginners (short-term) and advanced (long-term) riders with cerebral palsy on their whole mobility. The study involved 15 subjects (two girls and eleven boys). The subjects were aged from 3 to 19 years (8.73 years ± 5.85). All of the subjects had been diagnosed with a spastic form of cerebral palsy. The duration of the participation differed as follows: the advanced subjects had been riding for 1-4 years (2.66 years ± 1.16), while the beginners have been riding for two weeks (10 sessions). Group I (advanced riders) consisted of eight subjects (7 boys and 1 girl) who had therapy sessions regularly once a week and differed only in terms of the duration of their participation in the experiment. Group II (beginners) consisted of seven children (1 girl and 6 boys) who participated in only 10 riding sessions. All of the subjects were assessed according to the Gross Motor Function Measure (GMFM) and Gross Motor Function Classification System for CP (GMFCS) both before the investigation and after it. *Conclusions*. Ten riding lessons did not have an influence on the beginner riders with cerebral palsy gross motor functions and their gross motor function level did not change. However, in half of the advanced riders with cerebral palsy, the gross motor functions significantly improved. Meanwhile, the level of the performance of the gross motor skills in the four advanced riders increased, but this difference was not statistically significant.

## 1. Introduction

Cerebral palsy (CP) is a developmental disorder that affects the body position and movement, and that results in imbalance and muscles weakness due to the immaturity of parts of the brain that control activity and movements of the muscles. It is the most common cause of motor disabilities in children [1, 2]. The impairment is located in the part of the brain responsible for movements and sends the wrong signals to the muscles, causing the muscle tone to change so that those muscles become very tight or sag [2].

In the rehabilitation of children with CP, alternative and nontraditional therapies are used in parallel with traditional therapies. One of these is therapeutic horse riding. It is believed that while riding, a lateral flexing and extension of the rider's waist occurs, together with rotation, which reduces the muscle spasticity in the waist, pelvis, and lower limbs. The spasticity decreases when the horse's limbs are bent, retract, and swivel outward at the same time. Such rhythmic movements in three directions improve the tone of the pelvic and torso muscles [3, 4], as well as the thigh adductors muscles [5, 6].

Recently, a lot of research has been performed on the benefits of riding for the disabled. In the literature, we found works with positive assessments of hippotherapy (riding therapy) on children with cerebral palsy for its effects on the gait [7–11], energy expenditure [9], motor functions [9, 12–15], and muscle symmetry [5, 6]. However, we found no data in the literature on the short- and long-term effects of riding for children CP on the gross and fine motor functions. It is believed that exercises performed on unstable surfaces (e.g., when riding a horse) stimulate the neuromuscular control, stabilise the joints, and increase proprioception, as well as muscle control and its coactivation [16].

**The Hypothesis.** We believe that the movements of the horse's hind limbs in three spatial directions at the same time cause response movements in the rider that promote the general development of motor functions.

**The Aim. **It is to evaluate the effects of riding in beginners (short-term) and advanced (long-term) riders with common motility problems resulting from cerebral palsy.

We conducted our research in accordance with the convention of the human rights and dignity in medicine approved on 19 November 1996 (The Convention of Human Rights and Biomedicine) (Rodgers and Bousingen, 2001). The subjects and their parents/guardians were introduced to the purpose of our investigation, as well as the methods, procedures, and possible inconveniences. The Vilnius Regional Committee on Biomedical Research Ethics (No. 158200-11-069-31) issued the permit for biomedical research.

## 2. Materials and Methods

### 2.1. Materials

The study took place at the Kurtuvėnai Regional Park Riding Service Centre. The study involved 15 subjects (two girls and eleven boys) who were aged 3 to 19 years (8.73 years ± 5.85). All of the subjects were diagnosed with a spastic form of cerebral palsy, where only the type differed: nine subjects had spastic diplegia; three had dyskinesis; and two had tetraparesis (see Table 1). The duration of the participation in riding activities was also different: for the advanced subjects it ranged from 1 to 4 years (2.66 years ± 1.16), while for the beginners it consisted of only two weeks (10 sessions). Group I (the advanced riders) consisted of eight subjects (7 boys and 1 girl) who regularly participated in horse therapy sessions once a week, although the duration of their participation in this experiment differed. Group II (the beginner riders) consisted of seven children (1 girl and 6 boys) who only participated in 10 continuous riding sessions.

It should be noted that, in this study, the beginner riders were identified as those individuals who had never participated in riding lessons before. These riders were given ten riding therapy sessions. Meanwhile, the advanced riders in this study were identified as individuals who had been attending a regular riding therapy programme for more than one year (see Table 1).

### 2.2. Methods

For the evaluation of the gross motor functions (GMFM:* Gross Motor Function Measure)* the following variables were observed: lying and rolling (A); sitting (B); crawling and kneeling (C); standing (D); walking, running, and jumping (E). The subjects were evaluated in terms of their level of the gross motor functionalities (GMFCS:* Gross Motor Function Classification System for CP). *For Group I (the advanced riders), the tests results were recorded before, during and at the end of the study, while for Group II (the beginner riders) the results were recorded before and after the study.

We used MS Office EXCEL 2003 for the data analysis. We evaluated the arithmetic average, ± standard error, and the relative values. The reliability of the results was evaluated using the criteria of Student's* t-test*.

## 3. Results

**Figure 1:**
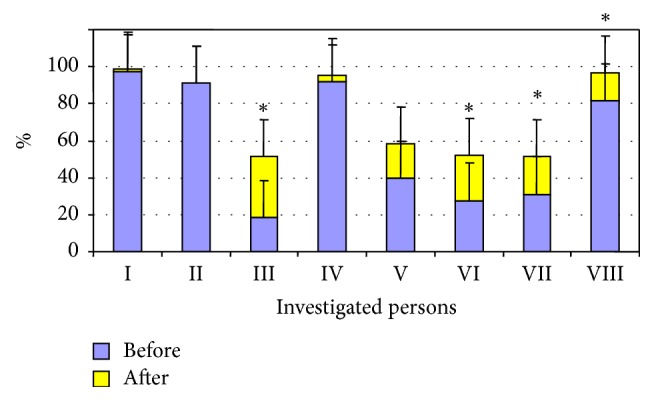
GMFM of the advanced riders before and after the examination (%).* Note.* Before: before the examination; after: after the examination; *∗* p<0.05 in comparison with the results before the study.

Before the study, the subjects were evaluated in terms of their gross motor functions (GMFM) (see Table 2) and the level of the Gross motor function classification system (GMFCS) (see Table 3). The advanced riders participated in riding sessions for an average of 3.25 ± 1.64 years. Only one subject's (second) gross motor functions remained unchanged during the whole of the study in quantitative terms, but the subject's thigh abduction and range of extension motion of the foot increased. Meanwhile the gross motor functions of the remaining subjects improved in comparison with the results that were recorded prior to when the research began. The GMFM of the first investigated person changed by 2.4%, but this difference was not statistically significant. The difference in the third participant's GMFM after the experiment (32.8%) was statistically significant (p<0.05) compared with the results obtained at the beginning of the study. Currently, this boy (6.5 years old) is able to balance on the horse while it is walking and he is learning to control the horse. For the fourth participant, the motor functions improved by 3%, but this difference was not statistically significant. At present, she is able to independently modify the direction of her movement and to stop and start the horse. The change in the fifth participant's GMFM was 18% overall, but this difference was not statistically significant. At the moment, this student is also able to independently change the direction of the horse's movement and to stop and start the horse. In comparing the results recorded at the beginning and the end of the GMFM study, a statistically significant difference (24.5%) was obtained for the sixth student (p <0.05) as well as for the overall change of the seventh student (p<0.05) (20%). For the eighth test participant, the total GMFM change was once again statistically significant (p<0.05) (15%) (see Figure 1).

**Figure 2:**
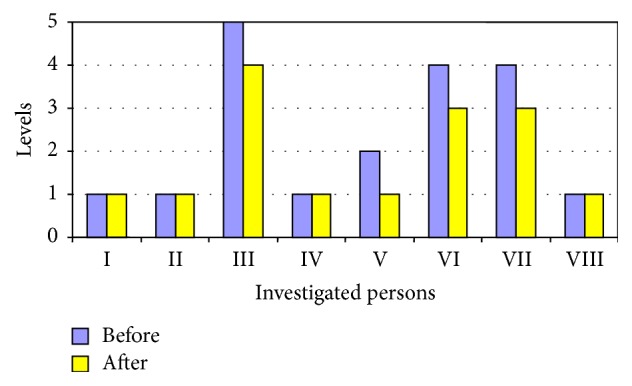
GMFCS level of the advanced riders during the experiment period.

For four riders in the advanced group, the level of the gross motor functionalities did not change during the experiment (the first, second, fourth, and eighth participants), while for the three of them (the third, fifth, sixth, and seventh participants) it increased (see Figure 2), but the change was not statistically significant.

**Figure 3:**
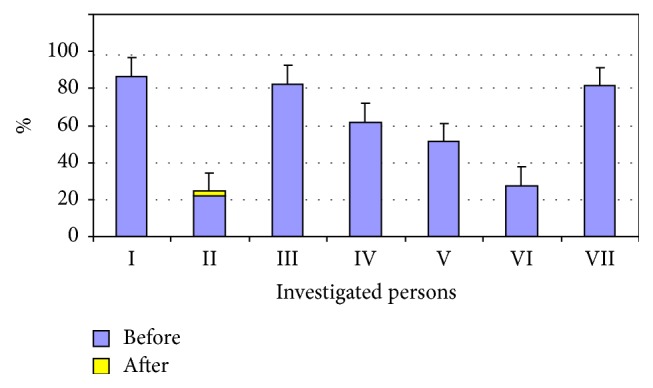
Gross motor functions (GMFM) before and after the examination in the group of beginner riders (%).

**Figure 4:**
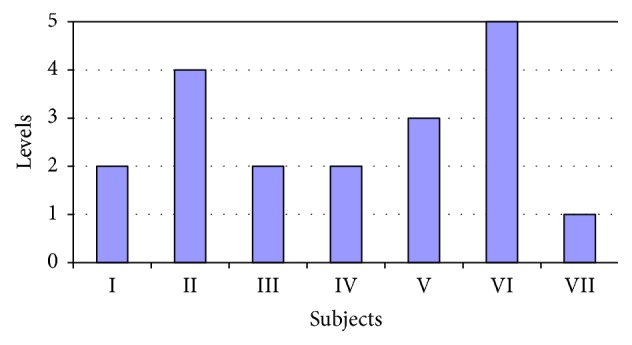
GMFCS levels before the experiment in the group of beginner riders.

All of the beginners took only 10 riding lessons. Comparing the results before and after the activities showed that the gross motor functions improved by 2.2% in only one of the investigated subjects (the second child), but this difference was not statistically significant. The gross motor functions of the other subjects remained unchanged after ten activity sessions (see Table 3, Figure 3). The levels of the gross motor functionalities also showed no change after the ten riding sessions in the beginners group (see Figure 4).

## 4. Discussion

The aim of this study was to determine the effects of short-term and long-term riding on the gross motor functions of children with cerebral palsy. To sum up the results of the motor function tests in the group of advanced riders, a statistically significant difference was obtained in a comparison of the gross motor functions investigated before and after the experiment in four of the eight subjects (p<0.05). Nonetheless, for others in the advanced riders group, the test results for the gross motor functions also improved but difference was not statistically significant. Furthermore, for one of the subjects (the second child) the gross motor functions did not change during the entire study. During the experiment and at the end of it, the number if gross motor functions increased only for four subjects (the third, fifth, and sixth children), while for the other subjects the gross motor functions remained unchanged, with only the quality and the mobility having improved (see Figures 3 and 4).

### 4.1. Does the Riding Therapy Improve the Gross Motor Functions for the Children with Cerebral Palsy?

Although a large number of articles can be found that analyse the effects of riding for the gross motor function changes in children with cerebral palsy, we did not find any study that compared the short-term and long-term effects of riding therapy. In the literature, it was found that at least 10 therapeutic riding sessions should be performed in order to reduce the spasticity [17]. According to our knowledge, this is the first research study to prove that ten riding sessions (a short-term effect) are not sufficient to improve the gross motor functions of children with cerebral palsy. Authors [15] also confirmed that after ten therapy sessions, the standardised test results did not show any changes in children with severe CP. This provides confirmation that, in order to develop new motor programmes and to teach new movements to children with cerebral palsy, 10 riding sessions are not enough. It is known that riding a horse can improve the head and trunk control [18], gait [10, 19, 20], balance, coordination, weight transfers, and posture [21] and can increase muscle strength, the range motion of the joints [22], and the gait parameters [23]. It is known that an internal model is made in the human motor cortex of the brain (the CNS mechanisms which manage to sense the power required to permit movement) that allows it to adjust to the new dynamic environment [24]. This is also confirmed by our research data, which shows that riding therapy that continues for a long time (i.e., from 1 to 2 times per week, all year round) helps a child to develop motor functionalities more accurately. For the subjects who participated in such a programme for a long term, the gross motor functions such as walking, running, and jumping showed a statistically significant improvement when the results before and after the experiment were compared (p<0.05). This can be explained by the following statement: pelvic mobility is initiated as a result of the rider's model that develops as a result of the horse's movements in a normal walking pattern. The repetition of the pelvic movements can help to restructure the child's CNS, which can in turn help to increase the functional activities. The literature states that the movements of the posterior legs, torso and pelvis of the horse are similar to those of the human torso, pelvis, and legs. It is known that the lateral displacement of a horse's pelvis is 4-5 cm, while that of a human is 7-8 cm, while the horse's joints can rotate around 8 degrees, and those of a human rotate 3-4 degrees. When the horse's posterior hoof rises from the ground and the horse's pelvis slips forward, the rider's torso is forced to move back. Conversely, when the horse's posterior hoof is placed on the ground and the horse's pelvis slips back, the rider's waist moves forward. While sitting on this dynamic surface, riding enables a person to develop torso coordination, balance, and the correct timing of the reaction. Regular initiation of muscle tension and release also promotes adaptive responses to develop the strategies and movements [14, 15]. In children with spastic diplegia, an imbalance of strength in the trunk muscles is caused because of a functional leg length difference, as well as impaired leg, pelvis and torso biomechanics, trunk muscle weaknesses, and sluggish postural (position) reflexes [25, 26]. The functional leg length difference is caused by asymmetrical calves and by muscle weakness of the quadriceps and hip extensors. Meanwhile, the spasticity of the lower limbs and the muscle group of the thigh adductors, asymmetry of the thigh adductors, and an increased coactivation of the abdominal and back muscles lead to a lack of pelvic and trunk dissociation. In the case of the named skeletal muscular deviations, the pelvic slope is disturbed, which travels to the waist and causes a disturbance and a strength imbalance in the torso muscles. An incorrect pelvis position is one of the largest problems for children with spastic diplegia because the pelvic bones are attached to the spine and the lower limbs transmit vertical forces between the kinetic chain. The pelvis also transmits the biomechanical problems of the lower limbs to the trunk. In the case of an impaired pelvis position in the frontal plane, the pelvis rotates at a time which is due to the medial tibial and lateral femoral rotation and the plantar flexion of the foot, which in turn initiates the rotation of the vertebrae [26].

Our study focused on the spastic form of cerebral palsy, which involves a pathological muscle tone and movement management, and this may lead to a pathological posture. Researchers [25, 26] confirmed that children with spastic diplegia are characterised by a pathological spinal geometry, sluggish postural (position) reflexes, and insufficient trunk control while sitting and/ or standing, so the quality of the posture while sitting and standing becomes impaired, and this has an impact on their daily activities and quality of life. These children also have difficulty in precontrolling the posture by making voluntary movements.

It is difficult to compare our research data with the results obtained by other authors, because the parameters such as the duration of the participation in the study and the frequency of the activities per week were different. In the published studies, the research lasted from 7 to 18 weeks. Furthermore, in four studies the riding sessions were held twice a week [9, 21, 22], while in the other three the sessions were conducted once a week [13, 14, 19]; and in one study they were held three times a week [23]. In all of the recent studies, the authors pointed out positive changes in the functionalities of the gross motor functions in both the quantitative and the qualitative aspects. Additionally, results of the study [13] confirmed that, during the riding time, (1) the three-dimensional reciprocal pelvic movements of the rider initiated by the movements of the walking horse resembled those of a healthy person while walking; (2) the gentle, rhythmic movements of the horse improved the muscle contraction, the stability of joints, and the weight transfers, as well as the balance and postural reactions; and (3) the hippotherapy improved the dynamic postural stabilisation, restored the disturbances, and helped with the preliminary and feedback control.

In our study, a statistically significant change was recorded in the gross motor functions of half of the group of advanced riders, while at the end of the experiment gross motor function levels had increased in the group of advanced subjects, but this difference was not statistically significant. The parents of the advanced riders also pointed out that although some of the subjects in the study did not exhibit a statistically significant difference in the quantitative aspects, the quality and mobility of the subjects had nonetheless improved; i.e., they were able to travel more independently, indicating an improvement in the physical capacity and energy consumption indicators. Their movements had become more precise, with the torso movement more straight forward. It has been confirmed that when the gross motor functions become better, the coordination of movements and the functional mobility of a person with cerebral palsy will also improve.

We cannot state that the long-term effects of riding are more effective than the short-term effects due to several factors. First of all, the riding time, frequency, and number of riders varied in our study. The beginner riders (who experienced a short-term effect) took part in the study for 2-3 weeks, while the advanced (long-term effect) riders participated in the study for a period of 3.25 ± 1.64 years. Secondly, it should be noted that at the time when the beginners participated in the riding classes they did not receive any other rehabilitation services. Meanwhile, the more advanced riders received rehabilitation services such as massage and kinesiotherapy at rehabilitation institutions in addition to the riding lessons. Third, the effects of the psychomotor development in the development process cannot be excluded, which could have affected the final outcome of the study.

## 5. Conclusions

Ten riding sessions did not influence the gross motor functions and the gross motor functionalities for the beginner riders with cerebral palsy. However, the gross motor functions significantly improved for one-half of the advanced riders with cerebral palsy. Meanwhile, the level of performance of the gross motor skills for the four advanced riders increased, but this difference was not statistically significant.

## Figures and Tables

**Table 1:** Characteristics of the advanced and beginner subjects.

**No.**	**Sex**	**Age**	**Diagnosis**	**Duration of participation in the study**
**Advanced**				

1	M	15 years	CP, spastic tetraparesis	3 years

2	M	19 years	CP, spastic dyskinesia	4 years

3	M	6 years	CP, spastic hemiparesis dyskinesia	4 years

4	F	15 years	CP, spastic dyskinesia	1.5 years

5	M	14 years	CP, spastic diplegia	5 years

6	M	4 years	CP, spastic diplegia	2 years

7	M	3.5 years	CP, spastic diplegia	1 year

8	M	3 years	CP, spastic diplegia	1 year

**Beginners**				

1	M	10 years	CP, spastic diplegia	10 lessons - 2 weeks

2	M	4 years	CP, spastic tetraparesis	10 lessons - 2 weeks

3	F	10 years	CP, spastic diplegia	10 lessons - 2 weeks

4	M	3.5 years	CP, spastic diplegia	10 lessons - 2 weeks

5	M	3 years	CP, spastic diplegia	10 lessons - 2 weeks

6	M	3 years	CP, spastic diplegia	10 lessons - 2 weeks

7	M	17 years	CP, spastic diplegia	10 lessons - 2 weeks

M: male; F: female.

**Table 2:** Changes in the gross motor functions (GMFM) for the advanced subjects at the different research stages.

**Investigated subject, Sex**	**GMFM evaluation areas** **Initial study (**%**)**					**GMFM evaluation areas** **Second study (**%**)**					**GMFM evaluation areas** **Third study (**%**)**				
	**A**	**B**	**C**	**D**	**E**	**A**	**B**	**C**	**D**	**E**	**A**	**B**	**C**	**D**	**E**

I (M)	100	100	100	94	94	100	100	100	94	97	100	100	100	**100**	97
II (M)	84	97	95	92	88	84	97	95	92	88	84	97	95	92	88
III (M)	60	30	2	0	0	**78** *∗*	**36** *∗*	**19** *∗*	**2** *∗*	**5** *∗*	**92** *∗*	**58** *∗*	**78** *∗*	**12** *∗*	**16** *∗*
IV (F)	92	98	100	82	72	**100**	98	100	87	72	100	98	100	87	**77**
V (M)	100	100	97	79	40	100	100	97	79	**58**	100	100	97	79	58
VI (M)	76	50	9	0	0	**92** *∗*	**65** *∗*	**40** *∗*	**7** *∗*	**9** *∗*	**96** *∗*	**73** *∗*	**64** *∗*	**10** *∗*	**18** *∗*
VII (M)	70	51	21	7	7	**98**	**68**	**40**	**10**	7	98	**73**	**64**	**10**	**11**
VIII (M)	100	90	83	43	16	100	**93** *∗*	**90** *∗*	**55** *∗*	**25** *∗*	100	**96**	**95**	**79**	**37**

M: male, F: female; GMFM: Gross motor functions measure; A: lying and turning over; B: sitting; C: crawling and kneeling; D: standing; E: walking, running, and jumping; M: male, F: female; **96** features improved *∗* p<0.05 in comparison with the first assessment.

**Table 3:** Change of the gross motor functions (GMFM) in the beginners group.

**Investigated students, Sex**	**GMFM evaluation areas** **Initial testing, **%					**GMFM evaluation areas** **Second study, **%				
	**A**	**B**	**C**	**D**	**E**	**A**	**B**	**C**	**D**	**E**
I (M)	98	96	85	84	79	98	96	85	84	79

II (M)	45	34	19	4	12	**47**	**35**	**23**	5	12

III (M)	100	100	92	66	55	100	100	92	66	55

IV (F)	92	85	83	28	20	92	85	83	28	20

V (M)	98	73	64	10	11	98	73	64	10	11

VI (M)	48	39	34	5	11	48	39	34	5	11

VII (M)	100	96	95	79	37	100	96	95	79	37

GMFM: Gross motor functions measure; A: lying and turning over; B: sitting; C: crawling and kneeling; D: standing; E: walking, running, and jumping; M: male, F: female; **96** features improved.

## References

[1] Miller F. (2007). *Physical therapy of cerebral palsy*.

[2] Campbell S. K., Vander Linden D. W., Palisano R. J. (2005). *Physical Therapy for Children*.

[3] Lechner H. E., Feldhaus S., Gudmundsen L. (2003). The short-term effect of hippotherapy on spasticity in patients with spinal cord injury. *Spinal Cord*.

[4] Lechner H. E., Kakebeeke T. H., Hegemann D., Baumberger M. (2007). The Effect of Hippotherapy on Spasticity and on Mental Well-Being of Persons With Spinal Cord Injury. *Archives of Physical Medicine and Rehabilitation*.

[5] Benda W., McGibbon N. H., Grant K. L. (2003). Improvements in Muscle Symmetry in Children with Cerebral Palsy after Equine-Assisted Therapy (Hippotherapy). *The Journal of Alternative and Complementary Medicine*.

[6] McGibbon N. H., Benda W., Duncan B. R., Silkwood-Sherer D. (2009). Immediate and Long-Term Effects of Hippotherapy on Symmetry of Adductor Muscle Activity and Functional Ability in Children With Spastic Cerebral Palsy. *Archives of Physical Medicine and Rehabilitation*.

[7] Encheff J. L. (2006). Comparison of muscular activity of the trunk and lower extremity muscles during normal ambulation versus horseback riding. *Pediatric Physical Therapy*.

[8] Encheff J. L. (2008). *Kinematic gait analysis of children with neurological impairments pre and post hippotherapy intervention, [Dissertation, thesis]*.

[9] McGibbon N. H., Andrade C.-K., Widener G., Cintas H. L. (1998). Effect of an equine-movement therapy program on gait, energy expenditure, and motor function in children with spastic cerebral palsy: A pilot study. *Developmental Medicine & Child Neurology*.

[10] McGee M. C., Reese N. B. (2009). Immediate effects of a hippotherapy session on gait parameters in children with spastic cerebral palsy. *Pediatric Physical Therapy*.

[11] Schwesig R., Neumann S., Richter D. (2009). Impact of therapeutic riding on gait and posture regulation. *Sportverletzung Sportschaden*.

[12] Brock B. (1989). *Therapy on Horseback: Psychomotor and Psychological Change in Physically Disabled Adults[Dissertation, thesis]*.

[13] Sterba J. A., Rogers B. T., France A. P., Vokes D. A. (2002). Horseback riding in children with cerebral palsy: Effect on gross motor function. *Developmental Medicine & Child Neurology*.

[14] Casady R. L., Nichols-Larsen D. S. (2004). The Effect of Hippotherapy on Ten Children with Cerebral Palsy. *Pediatric Physical Therapy*.

[15] Hamill D., Washington K. A., White O. R. (2007). The effect of hippotherapy on postural control in sitting for children with cerebral palsy. *Physical & Occupational Therapy in Pediatrics*.

[16] Andrade R., Araújo R. C., Tucci H. T., Martins J., Oliveira A. S. (2011). Coactivation of the shoulder and arm muscles during closed kinetic chain exercises on an unstable surface. *Singapore Medical Journal*.

[17] Cunha A. B., Novaes G. F., Rezende L. C. Therapeutic horseback riding results on the muscular tonus of lower limbs and motor performance in children with spastic cerebral palsy , XII International Congress of Therapeutic Riding.

[18] Shurtleff T. L., Standeven J. W., Engsberg J. R. (2009). Changes in Dynamic Trunk/Head Stability and Functional Reach After Hippotherapy. *Archives of Physical Medicine and Rehabilitation*.

[19] Winchester P., Kendall K., Peters H., Sears N., Winkley T. (2002). The effect of therapeutic horseback riding on gross motor function and gait speed in children who are developmentally delayed. *Physical & Occupational Therapy in Geriatrics*.

[20] Haehl V., Giuliani C., Lewis C. (1999). Influence of hippotherapy on the kinematics and functional performance two children with cerebral palsy. *Pediatric Physical Therapy*.

[21] Bertoti D. B. (1988). Effect of therapeutic horseback riding on posture in children with cerebral palsy. *Physical Therapy in Sport*.

[22] Low S., Collins G., Dhagat C., etal. (2005). Therapeutic horseback riding: its effects on gait and gross motor function in children with cerebral palsy. *Scientific and educational journal of therapeutic riding*.

[23] Honkavaara M., Rintala P. (2010). The influence of short term, intensive hippotherapy on gait in children with cerebral palsy. *European Journal of Adapted Physical Activity*.

[24] Stanislovaitienė J. (2008). *Control of accuracy and stability of muscle isometric and dynamic contractions in different conditions of motor system: of doctoral dissertation: biomedical sciences, biology(01B), physiology(B470)*.

[25] El-Meniawy G. H., Thabet N. S. (2012). Modulation of back geometry in children with spastic diplegic cerebral palsy via hippotherapy training. *Egyptian Journal of Medical Human Genetics*.

[26] Graham K. H. Botulinum Neurotoxins in the Management of Cerebral Palsy.

